# Are health sciences students prepared for pharmacovigilance? A nationwide study of knowledge and suspected adverse drug reaction reporting

**DOI:** 10.1186/s12909-026-10012-4

**Published:** 2026-07-29

**Authors:** Dorota Kopciuch, Julia Wach, Krzysztof Kus

**Affiliations:** Department of Pharmacoeconomics and Social Pharmacy, Poznan University of Medical Sciences, Rokietnicka 3 Street, Poznan, 60-806 Poland

**Keywords:** Pharmacovigilance, Suspected adverse drug reaction reporting, Health sciences education, Medical education, Patient safety

## Abstract

**Background:**

Pharmacovigilance and suspected adverse drug reaction (ADR) reporting are key components of patient safety and post-marketing drug surveillance. Spontaneous reporting systems concern suspected reactions, because causality is not established at the time of reporting. Despite their importance, suspected ADRs remain widely underreported, commonly due to limited knowledge, insufficient procedural competence, and inadequate training of healthcare professionals. Undergraduate education is therefore important for future pharmacovigilance capacity, yet comparative evidence across health sciences disciplines remains limited.

**Methods:**

A nationwide, cross-sectional survey was conducted among health sciences students enrolled in long-cycle or first- or second-cycle programmes in Poland between October 2024 and October 2025. An anonymous online questionnaire, distributed via academic and student networks, assessed pharmacovigilance knowledge, attitudes toward suspected ADR reporting, perceived barriers, and educational needs. Associations between categorical variables were analysed using Pearson’s chi-square or likelihood ratio tests, with effect sizes expressed as Cramér’s V. Multiple comparisons were adjusted using the Benjamini–Hochberg false discovery rate procedure.

**Results:**

A total of 648 complete questionnaires were analysed. Significant disparities in pharmacovigilance knowledge and regulatory competence were observed across fields of study. Pharmacy students demonstrated higher awareness, more accurate conceptual understanding, and greater ability to correctly identify the competent authority for suspected ADR reporting compared with medical, nursing/midwifery, and other health sciences students (all q < 0.001, large effect sizes). While attitudes toward reporting were generally positive, they were not consistently supported by adequate procedural knowledge, particularly outside pharmacy curricula. Pharmacovigilance competence showed a non-linear relationship with year of study, with lower levels observed among sixth-year students in selected disciplines. Key barriers, including lack of remuneration and reluctance to assume additional workload, were already apparent at the undergraduate stage. Support for expanding pharmacovigilance education was high across all disciplines.

**Conclusions:**

The findings indicate important gaps in pharmacovigilance preparedness among health sciences students, especially in procedural and regulatory aspects of suspected ADR reporting. Undergraduate curricula should include more consistent, longitudinal, and practically focused pharmacovigilance training.

**Supplementary Information:**

The online version contains supplementary material available at https://doi.org/10.1186/s12909-026-10012-4.

## Key Points

• Pharmacovigilance knowledge and suspected ADR reporting competence among health sciences students vary markedly by field of study.

• Pharmacy students demonstrate higher awareness and regulatory competence than students of other health-related disciplines.

• Positive attitudes toward suspected ADR reporting do not translate into adequate procedural knowledge, particularly outside pharmacy education.

• Pharmacovigilance competence does not increase linearly with year of study, with a notable decline among sixth-year students in selected disciplines.

• Students strongly support expanding pharmacovigilance education, indicating a need for mandatory, longitudinal, and practically focused training.

**Supplementary Information:**

The online version contains supplementary material available at https://doi.org/10.1186/s12909-026-10012-4.

## Introduction

Pharmacovigilance (PV) is a core component of patient safety and post-marketing drug surveillance. According to the World Health Organization, pharmacovigilance encompasses the science and activities concerned with the detection, assessment, understanding, and prevention of adverse effects or any other drug-related problems [1]. Spontaneous reporting systems remain essential for identifying rare, delayed, or population-specific safety signals after medicines are introduced into clinical practice [2].

A key methodological distinction is that spontaneous reporting concerns suspected adverse drug reactions (ADRs), because a causal relationship between the medicine and the reported event has not yet been formally established at the time of submission. For this reason, undergraduate education should prepare future healthcare professionals not only to understand pharmacovigilance conceptually, but also to recognise potentially reportable events and use the correct national reporting pathways.

Despite regulatory development and international guidance, underreporting of suspected ADRs remains a persistent challenge [3, 4]. Previous studies have identified insufficient knowledge, uncertainty about reporting procedures, lack of confidence, and ambiguity about professional responsibilities as important contributors to low reporting rates [5, 6]. These barriers may originate during undergraduate education, before students enter independent clinical practice.

Pharmacovigilance knowledge and reporting behaviour vary across professional contexts [7, 8], while patient-related reporting barriers and the need for interprofessional collaboration also shape the functioning of pharmacovigilance systems [9, 10]. Existing studies indicate that pharmacovigilance education and preparedness among healthcare students remain heterogeneous, with recurrent gaps in knowledge and practical reporting competence [3, 5, 6, 11–13]. However, comparative evidence within a single national educational context remains limited, especially when knowledge, attitudes, perceived barriers, and educational needs are assessed together.

Therefore, the aim of the present study was to assess pharmacovigilance knowledge, attitudes toward suspected ADR reporting, perceived barriers, and educational needs among Polish students of medicine, pharmacy, nursing, midwifery, and other health sciences. The study also examined whether these outcomes differed according to field of study, year of study, and size of academic city. The rationale was to identify actionable educational gaps that could inform undergraduate pharmacovigilance training and support future patient safety practice.

## Methods

### Study design and setting

A nationwide, cross-sectional, observational survey was conducted to assess knowledge of pharmacovigilance, attitudes toward suspected adverse drug reaction (ADR) reporting, perceived barriers, and educational needs among students enrolled in health sciences programmes in Poland.

Data were collected between October 2024 and October 2025 among students enrolled at medical universities and health sciences faculties across Poland, including both large academic centres and smaller university cities.

### Ethical considerations

The study protocol was reviewed by the competent Bioethics Committee, which issued a formal opinion confirming that the study did not meet the criteria of a medical experiment. The research was conducted pursuant to Bioethics Committee Resolution No. KB750/24, stating that ethical approval was not required due to the anonymous and non-interventional nature of the study.

Participation was voluntary and anonymous. All participants provided informed consent electronically prior to completing the questionnaire. No personally identifiable data were collected. The study was conducted in accordance with the Declaration of Helsinki and principles of responsible research conduct.

### Study population and recruitment

The study population comprised students enrolled in long-cycle or first- and second-cycle health sciences degree programmes, including medicine, pharmacy, nursing, midwifery, and other medical professions. Eligible participants reported being in the fourth, fifth, or sixth year of their educational pathway. In the Polish higher education system, medicine is a six-year long-cycle programme; pharmacy comprises 11 semesters (5.5 years), with the eleventh semester falling in the sixth academic year; nursing and midwifery comprise three-year first-cycle programmes followed by two-year second-cycle programmes; and programme duration within the other medical professions category varies by discipline. Year of study was recorded exactly as self-reported and was not post hoc standardised across programme structures. Thus, Year 6 did not refer exclusively to medicine: it also applied to pharmacy students in the eleventh semester and to respondents from other programmes with a structurally applicable sixth academic year. Nursing and midwifery do not include a standard sixth academic year; therefore, any self-reported Year 6 observations in this combined category were not interpreted or reported as a within-discipline Year 6 contrast. Because programme structures differ, the same year category should not be interpreted as an identical curricular stage or as equivalent exposure to pharmacovigilance across disciplines.

Data were collected using an online, self-administered questionnaire distributed nationwide through a multi-channel recruitment strategy, including student scientific societies, student government networks, social media platforms dedicated to medical students, and official institutional mailing lists at the Poznan University of Medical Sciences, following formal approval of university authorities. Participation was not financially incentivized. Only fully completed questionnaires were included in the analysis.

### Data collection instrument

The questionnaire was developed as a set of independent indicators rather than as a unidimensional psychometric scale. The initial item pool was prepared on the basis of pharmacovigilance frameworks, regulatory recommendations, and previous survey-based studies in pharmacovigilance education [1–3, 5, 6, 11–13]. Specifically, the WHO pharmacovigilance framework [1], EMA good pharmacovigilance practice recommendations [2], and published studies and reviews addressing pharmacovigilance knowledge, attitudes, reporting barriers, and education [3, 5, 6, 11–13] informed the scope and wording of the questionnaire items and response options. Face and content validity were assessed by three researchers with complementary expertise in pharmacology, pharmacovigilance-related education, survey methodology, and statistical analysis. The review focused on the relevance of each item to the study objectives, conceptual clarity, consistency with pharmacovigilance terminology, adequacy of response options, and suitability for students from different health sciences programmes. Discrepancies were discussed until consensus was reached, and wording was refined where needed to improve clarity and reduce ambiguity. Before the main data collection, the questionnaire was pilot-tested in a group of 100 students from the target population. The pilot phase assessed comprehensibility, logical flow, completion time, technical functionality of the online form, and whether the response categories were understandable and exhaustive. No major structural problems were identified; minor wording adjustments were introduced before nationwide distribution. The pilot responses were used only to assess and refine the instrument and were not included in the final analytical dataset. Because the questionnaire was designed to capture distinct knowledge, attitude, barrier, and educational-need indicators rather than a single latent construct, internal consistency coefficients such as Cronbach’s alpha were not calculated. The questionnaire was therefore treated as an expert-reviewed and pilot-tested survey instrument for descriptive and analytical purposes, rather than as a fully psychometrically validated scale.

### Study variables and bias control

Primary outcome variables included indicators of pharmacovigilance knowledge, attitudes toward suspected ADR reporting, correct identification of competent authorities, perceived barriers, and educational needs. Independent variables included field of study, year of study, and size of academic city. Sex and age were treated as descriptive variables.

Potential sources of bias were considered. Selection bias was mitigated through nationwide, multi-source recruitment. Information bias was limited by standardized questionnaire administration, and social desirability bias was reduced through anonymous participation. Due to the cross-sectional design, causal inferences were not attempted.

### Statistical analysis

Descriptive statistics were used to summarize participant characteristics. Categorical variables were reported as frequencies and percentages, and continuous variables as means with standard deviations.

Bivariate associations were assessed using Pearson’s chi-square (χ²) or likelihood ratio (G²) tests, with effect sizes expressed as Cramér’s V. To account for multiple comparisons, p-values were adjusted using the Benjamini–Hochberg false discovery rate (FDR) procedure, and q-values were reported. Statistical significance was defined as q < 0.05.

To identify independent predictors of pharmacovigilance competence, multivariable logistic regression models were constructed for three primary outcomes: (1) self-declared knowledge of pharmacovigilance, (2) correct definition of pharmacovigilance, and (3) correct identification of the competent suspected ADR reporting authority. Models were adjusted for field of study, year of study, city size, sex, and age. Adjusted odds ratios (aORs) with 95% confidence intervals (CIs) were reported.

Given non-linear patterns observed in bivariate analyses, an interaction term between field of study and year of study was included. Attitudinal variables were not modelled multivariably due to quasi-complete separation. All analyses were performed using R.

## Results

### Characteristics of the study population

A total of 648 complete questionnaires were included in the analysis. The study population was predominantly female (59.7%), with a mean age of 24.0 years (SD = 1.2). Most respondents studied in large academic centres (> 500,000 inhabitants; 70.5%). Pharmacy students constituted the largest subgroup (36.1%), followed by medicine (31.8%), nursing/midwifery (23.0%), and other medical professions (9.1%). Fifth-year students represented nearly half of the sample (44.6%), while fourth- and sixth-year students accounted for 24.4% and 31.0%, respectively (Table 1).

**Table 1:** Characteristics of the study population

Characteristic	Category / value	Overall *n* (%)
Sex	Female	387 (59.7)
	Male	261 (40.3)
Age, years	Mean (SD)	24.0 (1.2)
	Median (IQR)	24 (23–25)
City size	> 500,000 inhabitants	457 (70.5)
	251,000–500,000 inhabitants	160 (24.7)
	≤ 100,000 inhabitants	16 (2.5)
	101,000–250,000 inhabitants	15 (2.3)
Field of study	Pharmacy	234 (36.1)
	Medicine	206 (31.8)
	Nursing / Midwifery	149 (23.0)
	Other medical profession	59 (9.1)
Year of study	Year 5	289 (44.6)
	Year 6	201 (31.0)
	Year 4	158 (24.4)

### Pharmacovigilance knowledge across fields of study

Clear and statistically significant differences in pharmacovigilance knowledge were observed between fields of study (Table 2). Overall, 54.5% of respondents declared that they knew what pharmacovigilance is. This proportion was higher among pharmacy students (92.7%) than among medical students (38.8%), students of other medical professions (32.2%), and nursing/midwifery students (24.8%). The association was highly significant (χ² = 223.0, q < 0.001) and had a very large effect size (Cramér’s V = 0.587), indicating marked differences between disciplines.

**Table 2:** Key differences in pharmacovigilance knowledge, attitudes and educational needs by field of study knowledge of pharmacovigilance

Knowledge of pharmacovigilance										
Key outcome	Overall (%)	Pharmacy	Medicine	Other medical profession	Nursing / Midwifery	Test	χ² / G²	df	Cramér V	q (BH)
Knows what pharmacovigilance is (%)	54.5	92.7	38.8	32.2	24.8	χ²	223.034	3	0.587	< 0.001
Correct definition of pharmacovigilance (%)	69.3	95.3	66.5	42.4	43.0	LR χ²	216.903	3	0.348	< 0.001

Attitudes and ADR reporting practice										
Key outcome	Overall (%)	Pharmacy	Medicine	Other medical profession	Nursing / Midwifery	Test	χ² / G²	df	Cramér V	q (BH)
suspected ADR reporting is necessary (%)	83.6	100.0	53.4	96.6	94.6	χ²	203.883	3	0.561	< 0.001
suspected ADR reporting is a professional duty (%)	81.0	99.6	48.5	84.7	95.3	χ²	213.951	3	0.575	< 0.001
Correct authority identified (%)	68.5	93.2	67.5	45.8	40.3	LR χ²	171.620	3	0.290	< 0.001

Barriers to ADR reporting										
Key outcome	Overall (%)	Pharmacy	Medicine	Other medical profession	Nursing / Midwifery	Test	χ² / G²	df	Cramér V	q (BH)
Lack of time (%)	89.8	-	93.7	-	-	χ²	10.123	3	0.125	0.055
No additional remuneration (%)	55.6	70.9	73.8	23.7	18.8	χ²	155.924	3	0.491	< 0.001
Reluctance to perform extra work (%)	73.8	88.0	68.0	71.2	60.4	χ²	42.156	3	0.255	< 0.001

Educational needs and system improvements										
Key outcome	Overall (%)	Pharmacy	Medicine	Other medical profession	Nursing / Midwifery	Test	χ² / G²	df	Cramér V	q (BH)
Mandatory classes during studies (%)	91.4	91.0	94.7	79.7	91.9	χ²	13.168	3	0.143	0.017
Elective classes (%)	75.0	77.4	78.2	59.3	73.2	χ²	9.788	3	0.123	0.058
PV-specific training courses (%)	88.9	90.2	91.3	83.1	85.9	χ²	4.942	3	0.087	0.386

Similar differences were found for the ability to correctly define pharmacovigilance. While nearly all pharmacy students provided a correct definition (95.3%), the proportion was lower among medical students (66.5%) and particularly low among nursing/midwifery students (43.0%) and students of other medical professions (42.4%). These differences remained highly significant after correction for multiple testing (LR χ² = 216.9, q < 0.001; Cramér’s V = 0.348), suggesting gaps in conceptual understanding beyond simple awareness.

### Attitudes toward suspected ADR reporting and regulatory competence

Although attitudes toward suspected ADR reporting were generally positive, they varied considerably across disciplines (Table 2). Overall, 83.6% of respondents agreed that suspected ADR reporting is necessary and 81.0% considered it a professional duty. However, endorsement was almost universal among pharmacy students (100% and 99.6%, respectively), whereas only about half of medical students shared these views (53.4% and 48.5%). These differences were statistically significant with large effect sizes (Cramér’s V > 0.56), indicating that positive attitudes are strongly discipline-dependent.

Knowledge of regulatory responsibility showed a similar pattern. While 93.2% of pharmacy students correctly identified the competent authority for suspected ADR reporting, correct identification was observed in 67.5% of medical students, 45.8% of students of other medical professions, and 40.3% of nursing/midwifery students. This association was statistically significant (LR χ² = 171.6, q < 0.001) with a moderate effect size (Cramér’s V = 0.290), indicating an important regulatory knowledge gap outside pharmacy education.

### Perceived barriers to suspected ADR reporting

Perceived barriers to suspected ADR reporting were common across all disciplines but differed in relative importance (Table 2). Lack of time was reported by nearly 90% of respondents overall; however, this barrier did not retain statistical significance after correction for multiple comparisons, suggesting it represents a broadly shared constraint rather than a discipline-specific issue.

In contrast, lack of additional remuneration emerged as a strongly differentiating factor. This barrier was reported by 70.9% of pharmacy students and 73.8% of medical students, compared with 23.7% of students from other medical professions and 18.8% of nursing/midwifery students (χ² = 155.9, q < 0.001; Cramér’s V = 0.491). Reluctance to perform additional work showed a similar, although less pronounced, pattern (overall 73.8%; χ² = 42.2, q < 0.001), indicating that perceived systemic disincentives are already evident at the undergraduate level.

### Educational needs across disciplines

Support for expanding pharmacovigilance education was consistently high (Table 2). Mandatory pharmacovigilance classes during undergraduate studies were endorsed by 91.4% of respondents overall, with only modest differences between disciplines (χ² = 13.2, q = 0.017). Preferences for elective courses (75.0% overall) and pharmacovigilance-specific training programs (88.9% overall) did not differ significantly after correction, suggesting broadly shared educational expectations irrespective of field of study.

### Differences by year of study

Year of study was strongly associated with pharmacovigilance knowledge and attitudes (Table 3). Fifth-year students consistently demonstrated the highest levels of awareness and competence. For example, 76.5% of fifth-year students declared knowing what pharmacovigilance is, compared with 37.3% of fourth-year and 36.3% of sixth-year students (χ² = 101.8, q < 0.001; Cramér’s V = 0.396).

**Table 3:** Key differences in pharmacovigilance knowledge, attitudes and educational needs by year of study Knowledge of pharmacovigilance

Knowledge of pharmacovigilance										
Key outcome	Overall (%)	Pharmacy	Medicine	Other medical profession	Nursing / Midwifery	Test	χ² / G²	df	Cramér V	q (BH)
Knows what pharmacovigilance is	54.5	37.3	76.5	36.3	χ²	101.801	2	0.396	< 0.001	
Correct definition of pharmacovigilance	69.3	88.6	86.2	29.9	χ²	217.881	2	0.410	< 0.001	

Attitudes and ADR reporting practice										
Key outcome	Overall (%)	Year 4 (%)	Year 5 (%)	Year 6 (%)	Test	χ² / G²	df	Cramér V	q (BH)	
suspected ADR reporting is necessary	83.6	61.4	97.9	80.6	χ²	101.613	2	0.396	< 0.001	
suspected ADR reporting is a professional duty	81.0	60.1	96.2	75.6	χ²	91.927	2	0.377	< 0.001	
Correct authority identified	68.5	88.0	81.7	34.3	χ²	181.619	2	0.374	< 0.001	

Barriers to ADR reporting										
Key outcome	Overall (%)	Year 4 (%)	Year 5 (%)	Year 6 (%)	Test	χ² / G²	df	Cramér V	q (BH)	
No additional remuneration	55.6	67.7	59.2	40.8	χ²	28.734	2	0.211	< 0.001	
Reluctance to perform extra work	73.8	81.0	75.8	65.2	χ²	12.560	2	0.139	0.008	

Educational needs and system improvements										
Key outcome	Overall (%)	Year 4 (%)	Year 5 (%)	Year 6 (%)	Test	χ² / G²	df	Cramér V	q (BH)	
Willingness to expand PV knowledge	75.6	53.8	89.3	73.1	χ²	70.703	2	0.330	< 0.001	
Mandatory classes during studies	91.4	83.5	93.1	95.0	χ²	16.726	2	0.161	0.001	

A similar non-linear pattern was observed for correct definition of pharmacovigilance, which was high among fourth- and fifth-year students (88.6% and 86.2%, respectively) but dropped markedly among sixth-year students (29.9%). Correct identification of the competent reporting authority followed the same trend, decreasing from 88.0% in fourth-year students to 34.3% in sixth-year students (χ² = 181.6, q < 0.001). These findings suggest that final-year students may experience loss of previously acquired pharmacovigilance knowledge, lack of reinforcement, or insufficient longitudinal integration of pharmacovigilance teaching. However, this interpretation should be considered cautiously because the cross-sectional design does not allow direct assessment of within-student knowledge change over time.

### Differences by city size

City size was significantly associated with pharmacovigilance knowledge, attitudes, and perceived barriers (Table 4). Students studying in large academic centres (> 500,000 inhabitants) more frequently declared knowledge of pharmacovigilance (60.4%) and provided correct definitions (86.0%) compared with students from medium-sized cities, particularly those with 251,000–500,000 inhabitants, where correct definitions were reported by only 20.6%.

**Table 4:** Key differences in pharmacovigilance knowledge, attitudes and educational needs by city size

Knowledge of pharmacovigilance										
Key outcome	Overall (%)	City 101–250k (%)	City 251–500k (%)	City > 500k (%)	City ≤ 100k (%)	Test	χ² / G²	df	Cramér V	q (BH)
Knows what pharmacovigilance is	54.5	46.7	37.5	60.4	62.5	χ²	25.831	3	0.200	< 0.001
Correct definition of pharmacovigilance	69.3	73.3	20.6	86.0	75.0	LR χ²	239.129	3	0.355	< 0.001

Attitudes and ADR reporting practice										
Key outcome	Overall (%)	City 101–250k (%)	City 251–500k (%)	City > 500k (%)	City ≤ 100k (%)	Test	χ² / G²	df	Cramér V	q (BH)
suspected ADR reporting is necessary	83.6	100.0	93.1	79.2	100.0	LR χ²	30.145	3	0.189	< 0.001
suspected ADR reporting is a professional duty	81.0	86.7	90.0	77.0	100.0	LR χ²	21.357	3	0.163	< 0.001
Correct authority identified	68.5	86.7	28.1	81.2	93.8	LR χ²	176.095	3	0.308	< 0.001

Barriers to ADR reporting										
Key outcome	Overall (%)	City 101–250k (%)	City 251–500k (%)	City > 500k (%)	City ≤ 100k (%)	Test	χ² / G²	df	Cramér V	q (BH)
Lack of time	89.8	66.7	88.1	92.8	43.8	LR χ²	31.897	3	0.280	< 0.001
No additional remuneration	55.6	53.3	29.4	65.2	43.8	χ²	62.592	3	0.311	< 0.001
Reluctance to perform extra work	73.8	60.0	61.9	78.3	75.0	LR χ²	17.297	3	0.167	0.002
Insufficient knowledge of reporting procedure	84.0	40.0	80.0	87.1	75.0	LR χ²	20.963	3	0.207	< 0.001

Educational needs and system improvements										
Key outcome	Overall (%)	City 101–250k (%)	City 251–500k (%)	City > 500k (%)	City ≤ 100k (%)	Test	χ² / G²	df	Cramér V	q (BH)
Willingness to expand PV knowledge	75.6	80.0	86.2	71.3	87.5	LR χ²	17.067	3	0.156	0.003
Elective classes during studies	75.0	40.0	78.8	76.4	31.2	LR χ²	23.421	3	0.207	< 0.001
Mandatory classes during studies	91.4	66.7	91.9	93.0	62.5	LR χ²	18.928	3	0.215	0.001

Correct identification of the competent authority also varied substantially, ranging from 81.2 to 93.8% in the largest and smallest cities to 28.1% in medium-sized cities (LR χ² = 176.1, q < 0.001; Cramér’s V = 0.308). Perceived barriers, including insufficient knowledge of reporting procedures and lack of remuneration, showed significant contextual variation, reinforcing the role of educational environment and institutional exposure in shaping pharmacovigilance competence.

### Exposure to pharmacovigilance education and sources of knowledge

**Fig. 1:**
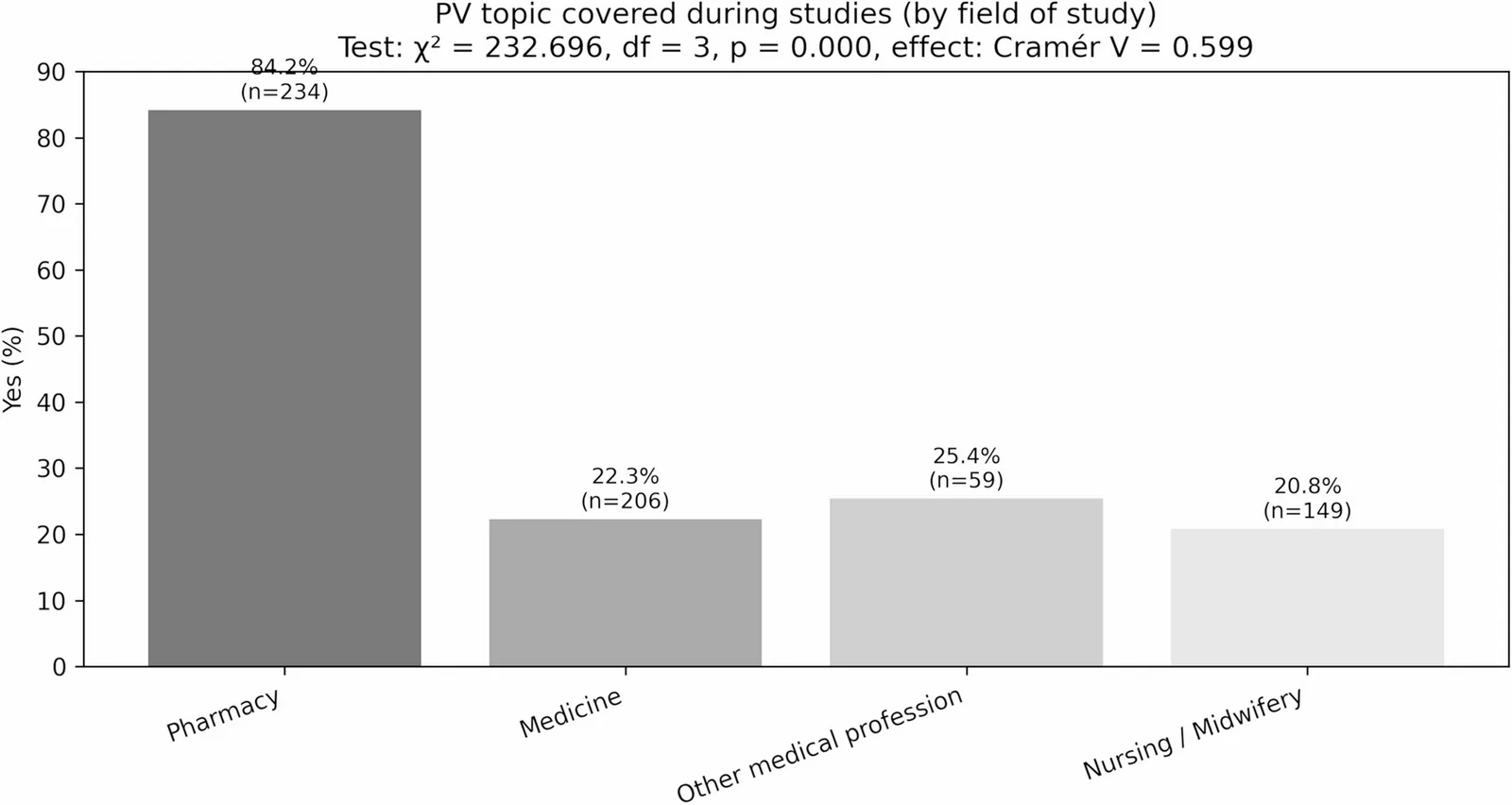
Pharmacovigilance topic covered during studies by field of study. Test statistics are provided in the title

**Fig. 2:**
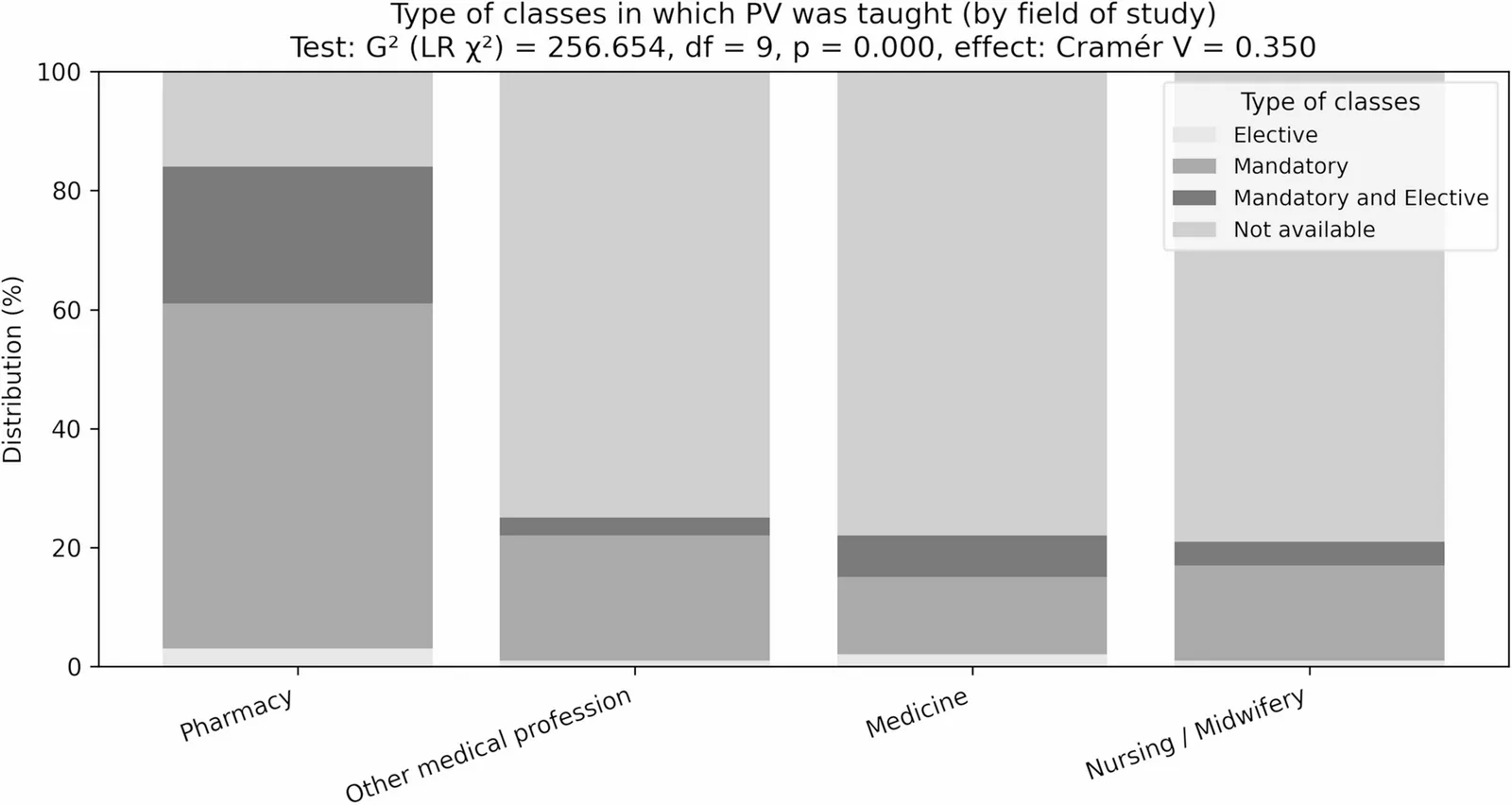
Type of classes in which pharmacovigilance was taught by field of study

As shown in Figs. 1 and 2, pharmacovigilance topics were most consistently covered during undergraduate education among pharmacy students, predominantly within mandatory courses, whereas exposure among other disciplines was more fragmented and often limited to selected classes.

**Fig. 3:**
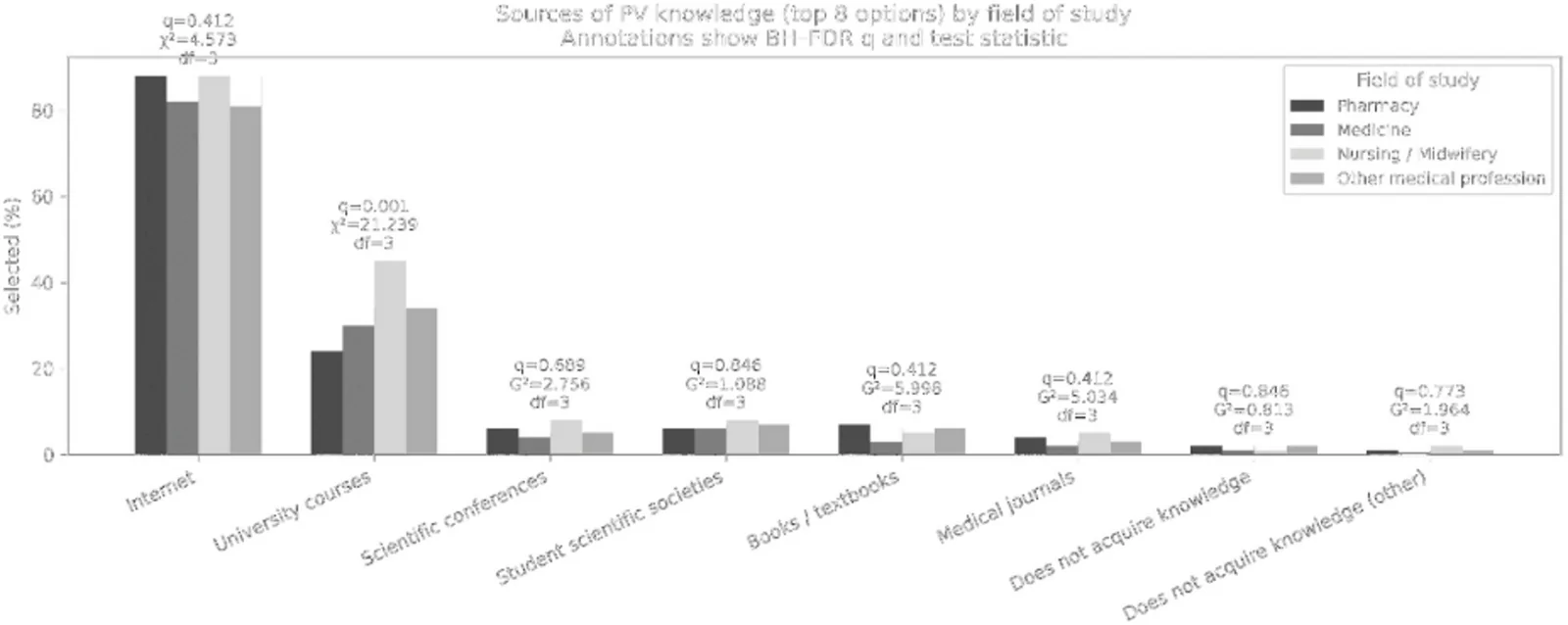
Sources of pharmacovigilance knowledge (top 8 options) by field of study. Annotations indicate q-values (BH) and χ²/G² statistics

**Fig. 4:**
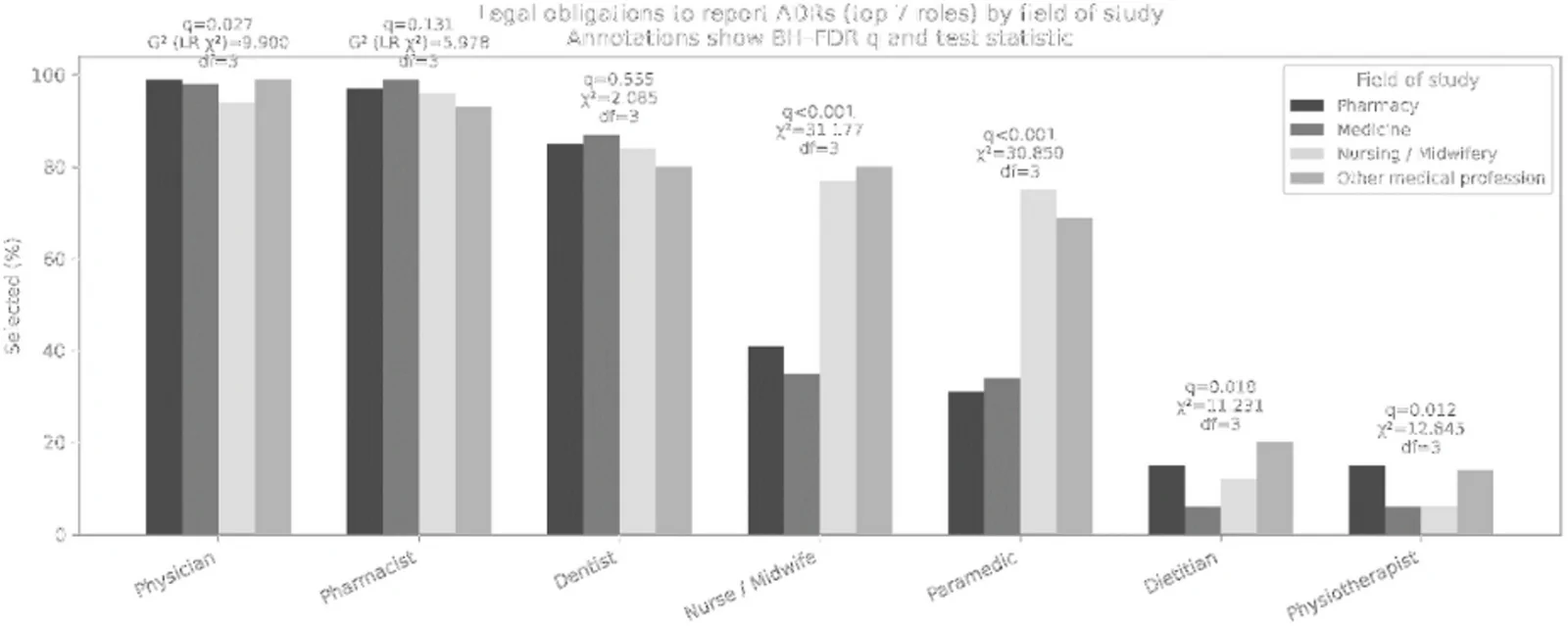
Legal obligations to report adverse drug reactions (top 7 professional roles) by field of study

Analysis of sources of pharmacovigilance knowledge (Fig. 3) demonstrated significant disciplinary differences, with pharmacy students relying primarily on formal academic instruction, while non-pharmacy students more frequently reported self-directed or informal sources. Awareness of legal obligations to report ADRs also varied by profession (Fig. 4), further underscoring disparities in regulatory preparedness across student groups.

### Multivariable predictors of pharmacovigilance competence

To determine whether the observed bivariate differences were independent of potential confounding factors, multivariable logistic regression models were performed for selected knowledge-based outcomes (Table 5).

**Table 5:** Multivariable predictors of pharmacovigilance knowledgeand regulatory competence (adjusted logistic regression)

Outcome	Predictor	aOR	95% CI	*p*-value
Knows what PV is	Year 6 vs. Year 5 (Pharmacy)	0.045	0.010–0.207	< 0.001
	Year 6 vs. Year 5 (Medicine)	0.801	0.292–2.196	0.666
	Year 6 vs. Year 5 (Other)	0.097	0.019–0.491	0.0048
Correct authority identified	Year 6 vs. Year 5 (Pharmacy)	0.013	0.002–0.088	< 0.001
	Year 6 vs. Year 5 (Medicine)	0.234	0.058–0.941	0.041
	Year 6 vs. Year 5 (Other)	0.089	0.011–0.707	0.022

Models adjusted for field of study, year of study, city size, sex, and age. Interaction term field × year included. Within-discipline Year 6 contrasts are not reported for nursing/midwifery because these programmes do not include a standard sixth academic year

After adjustment for field of study, year of study, city size, sex, and age, field of study remained the strongest independent predictor of pharmacovigilance competence across all primary outcomes.

### Interaction between field and year of study

A statistically significant interaction between field of study and year of study was identified for all three knowledge-based outcomes (*p* for interaction < 0.01), indicating that the association between year of study and pharmacovigilance competence differed across disciplines.

In adjusted models examining self-declared knowledge of pharmacovigilance, sixth-year students demonstrated significantly lower odds compared with fifth-year students within selected disciplines. Among pharmacy students, the odds of declaring pharmacovigilance knowledge were markedly reduced in Year 6 relative to Year 5 (aOR 0.045, 95% CI 0.010–0.207, *p* < 0.001). A similar decline was observed among students of other medical professions (aOR 0.097, 95% CI 0.019–0.491, *p* = 0.0048). In contrast, no statistically significant difference was identified among medical students (aOR 0.801, 95% CI 0.292–2.196, *p* = 0.666).

For correct identification of the competent authority, pharmacy students in Year 6 had lower adjusted odds compared with Year 5 (aOR 0.013, 95% CI 0.002–0.088, *p* < 0.001). Smaller but statistically significant declines were also observed among medical students (aOR 0.234, 95% CI 0.058–0.941, *p* = 0.041) and students of other medical professions (aOR 0.089, 95% CI 0.011–0.707, *p* = 0.022). A within-discipline Year 6 contrast was not reported for nursing/midwifery because these programmes do not include a standard sixth academic year.

### Reinterpretation of the year 6 pattern

Importantly, the previously observed non-linear decline in pharmacovigilance competence among final-year students was not uniform across all disciplines. The multivariable analysis indicates that this pattern is partly attributable to interaction effects between discipline and year of study rather than representing a generalized erosion of knowledge across the entire cohort.

Field of study remained independently associated with pharmacovigilance knowledge and regulatory competence even after full adjustment.

## Discussion

This nationwide study shows that pharmacovigilance knowledge, regulatory competence, and preparedness for suspected adverse drug reaction (ADR) reporting differ across health sciences students in Poland. The results point to undergraduate educational gaps, particularly outside pharmacy education. They should, however, be interpreted as evidence of educational preparedness rather than as direct evidence of future reporting behaviour.

The most consistent finding was the clear advantage observed among pharmacy students compared with students of medicine, nursing, midwifery, and other medical professions. Pharmacy students demonstrated higher awareness of pharmacovigilance, more accurate conceptual understanding, and better knowledge of suspected ADR reporting pathways and competent authorities. Comparable discipline-related differences and relatively stronger pharmacovigilance preparedness among pharmacy students have been reported in previous studies and systematic reviews [11–13]. Reviews of pharmacovigilance education also indicate that teaching across healthcare curricula remains heterogeneous and often requires greater standardisation, practical application, and longitudinal reinforcement [5, 12, 14].

An important finding is the discrepancy between favourable attitudes toward suspected ADR reporting and regulatory or procedural competence. Most respondents recognised reporting as necessary and professionally important, yet many were unable to identify the competent authority or reporting pathway correctly. From a patient-safety perspective, positive attitudes alone are unlikely to translate into effective participation in spontaneous reporting systems if graduates do not know what should be reported, where to report it, and how to complete the process. Similar attitude–knowledge discrepancies have been reported among healthcare students and future physicians [3, 11, 13, 14].

The study also shows that barriers commonly reported by practising clinicians—such as lack of remuneration, administrative burden, and reluctance to perform additional work—are already visible among students. This suggests that perceptions of suspected ADR reporting may be shaped before independent professional practice begins. Undergraduate education should therefore present pharmacovigilance not as an optional administrative task, but as a routine element of clinical reasoning, medicine safety, and professional responsibility. These observations are consistent with previous evidence identifying workload, organisational constraints, limited procedural support, and uncertainty about reporting as determinants of underreporting [4, 7, 15, 16].

One of the most original findings was the lower pharmacovigilance competence observed among sixth-year students in several analyses. This pattern may suggest loss of knowledge, insufficient reinforcement, or non-longitudinal teaching, particularly when pharmacovigilance is concentrated in isolated modules rather than revisited throughout the curriculum. At the same time, the multivariable models indicate that this decrease was not uniform across all fields of study and was partly modified by the interaction between discipline and year of study. Therefore, the Year 6 pattern should be interpreted as a signal of potential curricular discontinuity rather than as definitive evidence of knowledge erosion in every student group. Comparable concerns regarding insufficient pharmacovigilance preparedness among future doctors have been reported in a nationwide study [14].

Previous studies have documented variation in pharmacovigilance knowledge across professional and institutional contexts [7, 8], but direct evidence concerning academic city size is limited. In the present study, students in large academic centres generally demonstrated higher levels of knowledge and regulatory awareness than students in some smaller or medium-sized centres. However, curricula, staffing, educational resources, and institutional patient-safety activities were not directly measured. The mechanisms underlying this association therefore cannot be established, and the finding should be regarded as exploratory rather than as evidence that city size itself determines pharmacovigilance preparedness.

These results have practical implications for undergraduate education. Pharmacovigilance training should be mandatory, longitudinal, and focused on application across health sciences programmes. Teaching should include case-based recognition of suspected ADRs, hands-on completion of reporting forms, identification of competent national authorities, and discussion of how spontaneous reports contribute to signal detection and patient safety. Interprofessional education may be particularly useful, because suspected ADR reporting is a shared responsibility across healthcare professions. Educational interventions, particularly workshops and practice-based training, have been shown to improve pharmacovigilance knowledge and ADR reporting outcomes [17, 18].

The main strengths of this study include its nationwide scope, inclusion of several health sciences disciplines, assessment of knowledge, attitudes, barriers, and educational needs within the same survey, and statistical adjustment for multiple comparisons. The use of effect sizes and multivariable models also supports more cautious interpretation of the observed differences. These strengths help to identify educational priorities, although they do not eliminate the limitations inherent to the cross-sectional and questionnaire-based design.

## Conclusion

This nationwide study identified important gaps in pharmacovigilance preparedness among health sciences students in Poland, with pronounced differences between disciplines. Pharmacy students showed higher knowledge and regulatory competence, whereas students of medicine, nursing, midwifery, and other medical professions were less consistently prepared for suspected ADR reporting.

The findings indicate that favourable attitudes toward pharmacovigilance do not necessarily translate into practical procedural competence. The observed decrease among sixth-year students in selected analyses may reflect insufficient longitudinal reinforcement of pharmacovigilance education, although causal interpretation is limited by the cross-sectional design.

Undergraduate pharmacovigilance education should be strengthened through mandatory, longitudinal, and application-focused training. Particular emphasis should be placed on recognition of suspected ADRs, correct reporting pathways, regulatory responsibilities, and the role of spontaneous reports in patient safety. Such educational improvements may support more competent future participation in pharmacovigilance systems, although longitudinal and interventional studies are needed to confirm their effect on actual reporting behavior.

### Limitations

Several limitations of this study should be acknowledged. First, the cross-sectional design precludes causal inference and does not allow direct assessment of individual changes in pharmacovigilance competence over time; therefore, the lower competence observed among sixth-year students should be interpreted as an association rather than proof of knowledge loss. Second, data were self-reported and may be subject to recall bias, response bias, or social desirability bias, although anonymous participation likely reduced this risk. Third, participation was voluntary and recruitment was conducted through academic and student networks, so selection bias cannot be excluded. The sample also included a relatively high proportion of pharmacy students and students from large academic centers, which may limit the generalizability of some findings. Additionally, because year of study was self-reported and not standardised across programme structures, comparisons by year may partly reflect differences in programme composition rather than progression within the same curriculum. The absence of a standard sixth academic year in nursing and midwifery also precluded interpretation of a Year 6 versus Year 5 contrast within that combined group.

Fourth, the questionnaire was an original instrument designed to assess independent categorical indicators of pharmacovigilance preparedness rather than a unidimensional psychometric scale. Expert review and pilot testing in 100 students were performed to improve face validity, content validity, clarity, feasibility, and technical functionality. However, no formal content-validity index, test-retest reliability assessment, factor analysis, or full psychometric validation was conducted. This should be considered when interpreting the findings, particularly because the instrument was not intended to generate a single composite pharmacovigilance competence score. Fifth, the study assessed knowledge, attitudes, perceived barriers, and educational needs, but did not measure actual suspected ADR reporting behavior in clinical practice. Future longitudinal and interventional studies should evaluate whether structured pharmacovigilance teaching improves practical reporting competence and subsequent participation in spontaneous reporting systems.

## Supplementary Information

Supplementary Material 1.

## Data Availability

The datasets generated and/or analysed during the current study are available from the corresponding author upon reasonable request. Public deposition was not performed because the dataset contains survey responses from students and may include indirectly identifiable information when combined across demographic and educational variables.

## Abbreviations

ADR: Adverse drug reaction
aOR: Adjusted odds ratio
BH: Benjamini–Hochberg
CI: Confidence interval
FDR: False discovery rate
G²: Likelihood ratio chi-square statistic
PV: Pharmacovigilance
χ²: Pearson’s chi-square statistic
