# Supplementary material for: Are health sciences students prepared for pharmacovigilance? A nationwide study of knowledge and suspected adverse drug reaction reporting
Source: BMC Med Educ. 2026 Jul 29;26:1721. doi: 10.1186/s12909-026-10012-4 (PMC13700308; doi:10.1186/s12909-026-10012-4)
Supplement: Supplementary file 1 [file 12909_2026_10012_MOESM1_ESM.docx]

Supplementary File 1. Survey questionnaire

**Are Medical and Health Sciences Students Prepared for Pharmacovigilance? A Nationwide Study of Knowledge and Adverse Drug Reaction Reporting**

*Note: The survey was administered in Polish. An English translation is provided below for reviewer assessment. Single-choice questions require one answer; multiple-choice questions allow selecting more than one option.*

# English version

**Q1. Sex**

*Type: Single choice*

- ☐ Female
- ☐ Male
- ☐ Prefer not to say

**Q2. Age (years)**

*Type: Open-ended*

Answer: ________________________________

**Q3. Size of the city where you study**

*Type: Single choice*

- ☐ Rural area / village
- ☐ City up to 100,000 inhabitants
- ☐ City 101,000 to 250,000 inhabitants
- ☐ City 251,000 to 500,000 inhabitants
- ☐ City over 500,000 inhabitants

**Q4. Field of study**

*Type: Single choice*

- ☐ Medicine
- ☐ Pharmacy
- ☐ Nursing / Midwifery
- ☐ Other medical profession
- ☐ Other (please specify): ________________________________

**Q5. Year of study**

*Type: Single choice*

- ☐ Year 4
- ☐ Year 5
- ☐ Year 6

**Q6. Do you know what pharmacovigilance is?**

*Type: Single choice*

- ☐ Yes
- ☐ No

**Q7. Select the correct definition of pharmacovigilance**

*Type: Single choice*

- ☐ The science and activities related to detecting, monitoring, assessing, and preventing adverse reactions associated with the use of medicinal products.
- ☐ A patient program focused on correct medicine use to avoid any adverse reactions.
- ☐ A hospital-only system for monitoring and assessing adverse reactions.
- ☐ A set of procedures aimed solely at reducing the costs of adverse reactions.

**Q8. Select the correct assumption/goal of pharmacovigilance**

*Type: Single choice*

- ☐ Ensuring the safety of medicinal products.
- ☐ Collecting data on adverse drug reactions.
- ☐ Assessing the costs associated with adverse drug reactions.
- ☐ Increasing medicine sales by improving marketing strategies.

**Q9. Where should adverse drug reactions be reported in Poland?**

*Type: Single choice*

- ☐ Office for Registration of Medicinal Products, Medical Devices and Biocidal Products (URPL).
- ☐ The manufacturer of the medicinal product.
- ☐ National Health Fund (NFZ).
- ☐ Local sanitary inspectorate.

**Q10. Select the professions that are legally obliged to report adverse drug reactions**

*Type: Multiple choice*

- ☐ Physician
- ☐ Dentist
- ☐ Pharmacist
- ☐ Nurse / Midwife
- ☐ Paramedic
- ☐ Other (please specify): ________________________________

**Q11. During your studies, have you been taught about pharmacovigilance?**

*Type: Single choice*

- ☐ Yes
- ☐ No

**Q12. If yes, pharmacovigilance was taught as**

*Type: Single choice*

- ☐ Mandatory classes
- ☐ Elective classes
- ☐ Both mandatory and elective classes
- ☐ Not applicable

**Q13. Would you like to expand your knowledge of pharmacovigilance during your education?**

*Type: Single choice*

- ☐ Yes
- ☐ No

**Q14. What are your sources of knowledge about pharmacovigilance?**

*Type: Multiple choice*

- ☐ University courses / studies
- ☐ Internet
- ☐ Scientific literature
- ☐ Medical journals
- ☐ Scientific conferences
- ☐ Student research clubs / scientific societies
- ☐ Work / internship / placement
- ☐ I am not interested in this topic / I have no knowledge
- ☐ Other (please specify): ________________________________

**Q15. Do you consider ADR reporting necessary for proper supervision of pharmacotherapy safety?**

*Type: Single choice*

- ☐ Yes
- ☐ No

**Q16. Do you consider ADR reporting your future professional duty?**

*Type: Single choice*

- ☐ Yes
- ☐ No

**Q17. Which factors may discourage healthcare professionals from reporting ADRs?**

*Type: Multiple choice*

- ☐ Lack of time
- ☐ Reluctance to perform additional work
- ☐ No additional remuneration
- ☐ Insufficient knowledge of the reporting procedure
- ☐ Other (please specify): ________________________________

**Q18. Which actions could increase knowledge of pharmacovigilance?**

*Type: Multiple choice*

- ☐ Mandatory pharmacovigilance classes during undergraduate studies
- ☐ Elective pharmacovigilance classes
- ☐ Pharmacovigilance-specific training courses
- ☐ Scientific conferences
- ☐ Student research clubs / scientific societies
- ☐ Internships / placements that include pharmacovigilance practice
- ☐ Mass media campaigns (e.g., radio/TV)
- ☐ Other (please specify): ________________________________

# Polish version (original)

**Q1. Płeć**

*Type: Jednokrotny wybór*

- ☐ Kobieta
- ☐ Mężczyzna
- ☐ Wolę nie odpowiadać

**Q2. Wiek (w latach)**

*Type: Otwarta odpowiedź*

Odpowiedź: ________________________________

**Q3. Wielkość miejscowości, w której studiujesz**

*Type: Jednokrotny wybór*

- ☐ Wieś
- ☐ Miasto do 100 tys. mieszkańców
- ☐ Miasto 101-250 tys. mieszkańców
- ☐ Miasto 251-500 tys. mieszkańców
- ☐ Miasto powyżej 500 tys. mieszkańców

**Q4. Kierunek studiów**

*Type: Jednokrotny wybór*

- ☐ Lekarski
- ☐ Farmacja
- ☐ Pielęgniarstwo/Położnictwo
- ☐ Inny zawód medyczny
- ☐ Other (please specify): ________________________________

**Q5. Rok studiów**

*Type: Jednokrotny wybór*

- ☐ 4
- ☐ 5
- ☐ 6

**Q6. Czy wie Pan/Pani, czym jest pharmacovigilance?**

*Type: Jednokrotny wybór*

- ☐ Tak
- ☐ Nie

**Q7. Wskaż poprawną definicję pharmacovigilance**

*Type: Jednokrotny wybór*

- ☐ Nauka i podejmowane aktywności obejmujące wykrywanie, monitorowanie, ocenę i zapobieganie działaniom niepożądanym związanym ze stosowaniem produktów leczniczych.
- ☐ Program dla pacjentów dotyczący poprawnego stosowania leków w celu uniknięcia jakichkolwiek działań niepożądanych.
- ☐ System monitorowania i oceny działań niepożądanych w warunkach szpitalnych.
- ☐ Zestaw procedur ukierunkowanych wyłącznie na redukcję kosztów działań niepożądanych.

**Q8. Wskaż poprawne założenie pharmacovigilance**

*Type: Jednokrotny wybór*

- ☐ Zapewnienie bezpieczeństwa stosowanych produktów leczniczych.
- ☐ Zbieranie danych na temat działań niepożądanych.
- ☐ Ocena kosztów związanych z działaniami niepożądanymi.
- ☐ Zwiększanie sprzedaży leków poprzez działania marketingowe.

**Q9. Gdzie zgłaszane są działania niepożądane w Polsce?**

*Type: Jednokrotny wybór*

- ☐ Urząd Rejestracji Produktów Leczniczych, Wyrobów Medycznych i Produktów Biobójczych (URPL).
- ☐ Producent produktu leczniczego.
- ☐ Narodowy Fundusz Zdrowia (NFZ).
- ☐ Powiatowa stacja sanitarno-epidemiologiczna.

**Q10. Zaznacz osoby prawnie zobligowane do zgłaszania działań niepożądanych**

*Type: Wielokrotny wybór*

- ☐ Lekarz
- ☐ Lekarz dentysta
- ☐ Farmaceuta
- ☐ Pielęgniarka/Położna
- ☐ Ratownik medyczny
- ☐ Other (please specify): ________________________________

**Q11. Czy na studiach została przedstawiona Panu/Pani tematyka pharmacovigilance?**

*Type: Jednokrotny wybór*

- ☐ Tak
- ☐ Nie

**Q12. Jeśli tak, zajęcia dotyczące pharmacovigilance były**

*Type: Jednokrotny wybór*

- ☐ Obligatoryjne
- ☐ Fakultatywne
- ☐ Obligatoryjne i fakultatywne
- ☐ Nie dotyczy

**Q13. Czy byłby/a Pan/Pani zainteresowany/a poszerzeniem wiedzy w zakresie pharmacovigilance w toku nauczania?**

*Type: Jednokrotny wybór*

- ☐ Tak
- ☐ Nie

**Q14. Skąd pozyskuje Pan/Pani wiedzę na temat pharmacovigilance?**

*Type: Wielokrotny wybór*

- ☐ Studia
- ☐ Internet
- ☐ Literatura
- ☐ Czasopisma o tematyce medycznej
- ☐ Konferencje naukowe
- ☐ Koła naukowe
- ☐ Praca/Staż
- ☐ Nie interesuję się tematem / Nie mam wiedzy
- ☐ Other (please specify): ________________________________

**Q15. Czy uważa Pan/Pani zgłaszanie działań niepożądanych za niezbędne dla poprawnego nadzoru nad bezpieczeństwem farmakoterapii?**

*Type: Jednokrotny wybór*

- ☐ Tak
- ☐ Nie

**Q16. Czy uważa Pan/Pani zgłaszanie działań niepożądanych za swój obowiązek związany z wykonywaniem w przyszłości zawodu medycznego?**

*Type: Jednokrotny wybór*

- ☐ Tak
- ☐ Nie

**Q17. Wskaż czynniki, które mogą zniechęcać przedstawicieli zawodów medycznych do zgłaszania działań niepożądanych**

*Type: Wielokrotny wybór*

- ☐ Brak czasu
- ☐ Niechęć do wykonywania dodatkowej pracy
- ☐ Brak dodatkowego wynagrodzenia
- ☐ Niewystarczająca wiedza na temat procedury zgłoszenia
- ☐ Other (please specify): ________________________________

**Q18. Jakie działania według Pana/Pani można wprowadzić w celu zwiększenia wiedzy na temat pharmacovigilance?**

*Type: Wielokrotny wybór*

- ☐ Zajęcia obligatoryjne w toku studiów
- ☐ Zajęcia fakultatywne na studiach
- ☐ Szkolenia pharmacovigilance
- ☐ Konferencje naukowe
- ☐ Koło naukowe
- ☐ Staż (praktyki)
- ☐ Kampanie informacyjne w mediach (np. radio/TV)
- ☐ Other (please specify): ________________________________
